# The application and progress of tissue engineering and biomaterial scaffolds for total auricular reconstruction in microtia

**DOI:** 10.3389/fbioe.2023.1089031

**Published:** 2023-09-22

**Authors:** Yeqian Huang, Hanxing Zhao, Yixi Wang, Siwei Bi, Kai Zhou, Hairui Li, Changchun Zhou, Yudong Wang, Wenqing Wu, Bo Peng, Jun Tang, Bo Pan, Baoyun Wang, Zhixing Chen, Zhengyong Li, Zhenyu Zhang

**Affiliations:** ^1^ Department of Burn and Plastic Surgery, West China Hospital, Sichuan University, Chengdu, China; ^2^ Department of Plastic Reconstructive and Aesthetic Surgery, West China Tianfu Hospital, Sichuan University, Chengdu, China; ^3^ National Engineering Research Center for Biomaterials, College of Biomedical Engineering, Sichuan University, Chengdu, China; ^4^ Plastic Surgery Hospital, Chinese Academy of Medical Sciences and Peking Union Medical College, Beijing, China

**Keywords:** microtia, auricular reconstruction, tissue engineering, biomaterial scaffold, 3D printing

## Abstract

Microtia is a congenital deformity of the ear with an incidence of about 0.8–4.2 per 10,000 births. Total auricular reconstruction is the preferred treatment of microtia at present, and one of the core technologies is the preparation of cartilage scaffolds. Autologous costal cartilage is recognized as the best material source for constructing scaffold platforms. However, costal cartilage harvest can lead to donor-site injuries such as pneumothorax, postoperative pain, chest wall scar and deformity. Therefore, with the need of alternative to autologous cartilage, *in vitro* and *in vivo* studies of biomaterial scaffolds and cartilage tissue engineering have gradually become novel research hot points in auricular reconstruction research. Tissue-engineered cartilage possesses obvious advantages including non-rejection, minimally invasive or non-invasive, the potential of large-scale production to ensure sufficient donors and controllable morphology. Exploration and advancements of tissue-engineered cartilaginous framework are also emerging in aspects including three-dimensional biomaterial scaffolds, acquisition of seed cells and chondrocytes, 3D printing techniques, inducing factors for chondrogenesis and so on, which has greatly promoted the research process of biomaterial substitute. This review discussed the development, current application and research progress of cartilage tissue engineering in auricular reconstruction, particularly the usage and creation of biomaterial scaffolds. The development and selection of various types of seed cells and inducing factors to stimulate chondrogenic differentiation in auricular cartilage were also highlighted. There are still confronted challenges before the clinical application becomes widely available for patients, and its long-term effect remains to be evaluated. We hope to provide guidance for future research directions of biomaterials as an alternative to autologous cartilage in ear reconstruction, and finally benefit the transformation and clinical application of cartilage tissue engineering and biomaterials in microtia treatment.

## 1 Introduction

Microtia is a congenital ear malformation that can develop as an isolated birth abnormality or one of the manifestations of other syndromes, which can be classified based on the severity of the deformity, from mild structural changes to complete absence of the auricle (Luquetti et al., 2012). Patients can present with stenosis of the external auditory canal, partial atresia or total atresia, and are often accompanied by hearing impairment (Siegert, 2003).

Microtia occurs in approximately 0.8–4.2 per 10,000 births (Yetman and Hormann, 2015). Current studies have indicated the association between microtia and certain genetic and environmental factors, but the etiology and cause of the extensive epidemic variability have not been thoroughly understood (Luquetti et al., 2012). The main surgical treatment for microtia is total auricle reconstruction. Current options for reconstructive materials broadly include autologous costal cartilage framework, implanted artificial material and auricular prostheses. The first two approaches can be placed subcutaneously or under a vascularized fascial flap and skin graft, while a prosthetic ear fixed on the skin is only applied in patients with severe injury or burn of the auricle, extensive scar resulting in insufficient skin volume, or those who failed in both former two procedures (Zhang et al., 2019a).

One of the basic factors of auricular reconstruction is the supporting framework under skin to maintain the fine anatomical structure of the ear. At present, the supporting framework/scaffold mainly includes the auricular framework carved from the patient’s autologous costal cartilage, the Medpor prefabricated framework, and the tissue-engineered cartilage auricular framework. Autologous costal cartilage transplantation has been the current gold standard treatment for auricular reconstruction, which has the advantages of easy engraving, no rejection, and low incidence of cartilage exposure (Wilkes et al., 2014). In 1920, Gillies first performed external ear reconstruction by embedding the sculpted autologous costal cartilage into the subcutaneous tissue of the mastoid region, which was the earliest auricular framework procedure for microtia (Berghaus and Toplak, 1986). Tanzer is considered one of the leading practitioners of autologous costochondral grafts in ear reconstruction. In 1959 he described a six-stage auricular reconstruction procedure and changed the initial 6-stage procedure to a 4-stage ear reconstruction method as the clinical practice progressed (Tanzer, 1959; Tanzer, 1967; Tanzer, 1978). In 1980, Brent modified the Tanzer method and simplified the operation into three stages. A series of improvements were proposed such as personalized design of ear scaffold and storage of wedge cartilage to support the auricle in later reconstruction to form a more three-dimensional structure (Brent, 1980; Brent, 1999; Brent, 2002). In 1993, Nagata further reduced the procedure to two stages and emphasized the use of superficial temporal fascia flaps and sectional skin. The Nagata method provides a more reliable covering tissue for the scaffold and the layered and reinforced stents also minimize the deformation, which has become one of the most widely used methods in auricle reconstruction (Nagata, 1994a; Nagata, 1994b; Nagata and Edgerton, 1994). Meanwhile, the three-stage tissue expansion method of auricle reconstruction with autologous costal cartilage has also developed rapidly. The tissue expander provides an additional skin flap to cover the cartilage framework without transplantation of the skin graft, and the scar can be invisible. The thinner skin can ensure a more natural appearance and skin color of the reconstructed auricle, especially in some elaborate subunit structures (Park, 2000).

Generally, the 6th, 7th, and 8th costal cartilages of the contralateral thorax were selected as the donor area for sculpting and reinforcing the main framework of the auricular framework (Bly et al., 2016). However, the procedure may cause variable cosmetic results, and costal cartilage harvest can also lead to donor-site defects such as pneumothorax, chest wall pain and chest wall scar (Humphries et al., 2022). Besides, the ideal timing of operation occurs at least from 6 years old in consideration of the development of costal cartilage for framework carving, while in adulthood, costal cartilage becomes calcified and gradually loses elasticity, which is not desirable for surgery (Zhang et al., 2019a). Medpor (Stryker, United States) is a prefabricated synthetic biocompatible porous polyethylene implant that can be custom shaped intraoperatively by heating or engraving (Ali et al., 2017; Jiang et al., 2021). Porous polyethylene is an inorganic, hydrophobic and non-resorbable material that can be utilized in facial reconstructive treatments. However, the main deficiencies of the Medpor auricular framework lie in the significant risk of implant extrusion, fracture, immunogenicity and infection compared to autologous tissue (Romo et al., 2006; Zhang et al., 2019b), which therefore is not commonly applied in ear reconstruction.

Therefore, with the need of an alternative to autologous cartilage, *in vitro* and *in vivo* study of biomaterial scaffolds, bioprinting technologies and cartilage tissue engineering have gradually become novel research hot points in auricular reconstruction research (Bichara et al., 2012). Tissue engineering aims to rebuild tissues and organs that can be surgically implanted by utilizing cells and biomaterials (Melgarejo-Ramírez et al., 2016), and the tissue-engineered cartilage possesses benefits including non-rejection, minimal or no invasiveness, the potential of large-scale production to ensure sufficient donors and controllable morphology (Chen and Liu, 2016). This review will mainly discuss the development, current application and research progress of cartilage tissue engineering in auricular reconstruction. We hope to provide guidance for future research directions of biomaterials and finally benefit the transformation and clinical practice in ear reconstruction treatment in microtia.

## 2 Tissue engineering in auricular cartilage tissue for ear reconstruction

**FIGURE 1:**
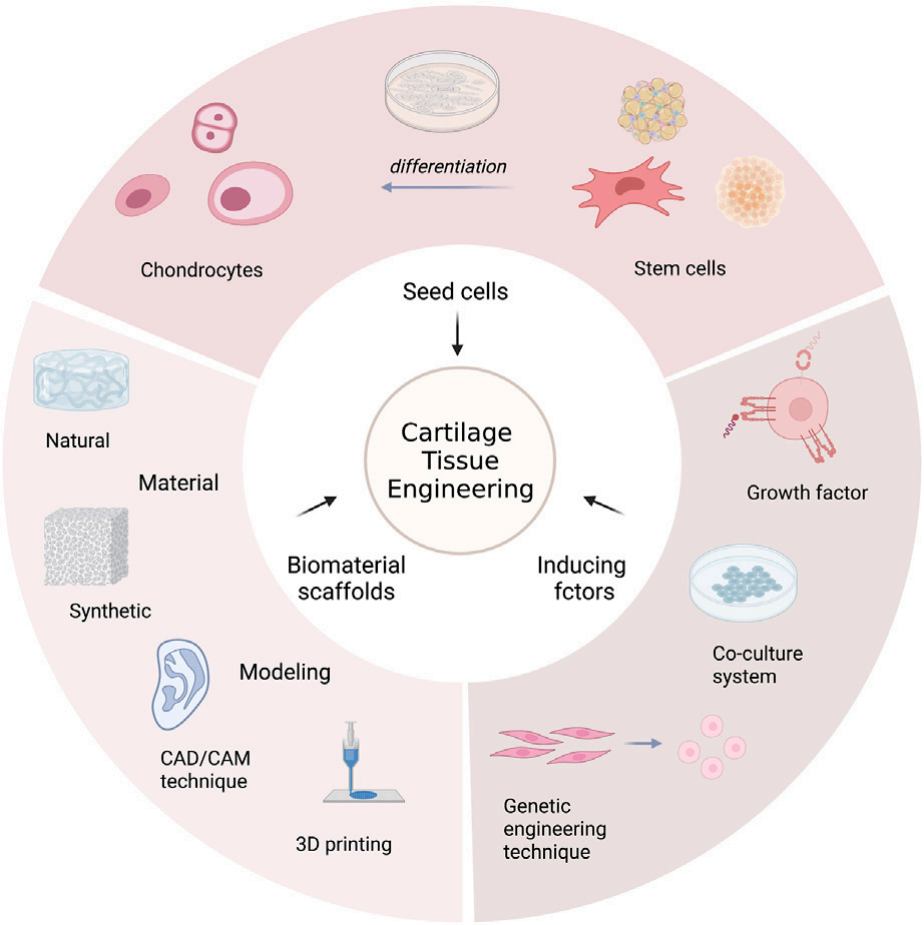
The overview of cartilage tissue engineering.

Tissue engineering technologies use a combination of cells, engineering, materials techniques, and appropriate biochemical and physicochemical parameters to generate different types of target tissues (Langer and Vacanti, 1993). In clinical practice, successful translation in the repair or replacement of portions or whole tissues has been reported in bone (Bhumiratana et al., 2016), cartilage (Whitney et al., 2017), blood vessels (Olausson et al., 2012), bladder (Atala et al., 2006), skin (Boyce and Lalley, 2018) *etc.*, which provides a novel direction for the auricular reconstruction in microtia. Cartilage tissue mainly consists of chondrocytes, extracellular matrix (ECM) and tissue fluid. The extracellular matrix (ECM) of cartilage is made up of collagen fibers (mostly type II collagen) supporting glycoproteins and proteoglycans with a protein core connected with glycosaminoglycan molecules such as hyaluronic acid (HA) and chondroitin sulfate (Hunziker et al., 2002). Exploration and challenges in generating a tissue-engineered cartilaginous framework mainly lie in the acquisition of optimal chondrogenic cell source and creation of a three-dimensional scaffold that the cells can grow upon, inducing factors for chondrogenesis and so on (Figure 1).

### 2.1 Seed cells

Tissue engineering utilizes progenitor cells that can proliferate and differentiate to obtain biological tissues. Currently, seed cell sources in relation to cartilage regeneration mainly include chondrocytes and stem cells such as bone-marrow-derived mesenchymal stem cells (BMSCs), adipose-derived stem cells (ADSCs), induced pluripotent stem cells (iPSCs), articular cartilage progenitor cells (ACPCs), *etc.*

#### 2.1.1 Chondrocyte

Chondrocytes isolated from a biopsy of patients’ autologous cartilage tissues continue to be the principal source of engineered cartilage, for its property of spontaneously secreting cartilage-specific matrix (Xu et al., 2005). Autologous chondrocytes can be obtained from articular hyaline cartilage, auricular elastic cartilage, nasoseptal elastic cartilage and costal hyaline cartilage. The biomechanical and metabolic characteristics of the resulting neocartilage are expected to vary depending on the source of cells employed, and whether the ectopic-derived chondrocytes can regenerate specific types of cartilage and achieve functional reconstruction remains uncertain. For auricular cartilage regeneration, the corresponding type of chondrocytes can be more accessible from the auricle, while it has also been proved that the chondrocytes obtained from nasal septum or auricle sites have higher proliferative activity (Tay et al., 2004; Zhang et al., 2014).

Since the initial attempt at *in vitro* culturing of the chondrocytes in the 1960s, the optimization of isolation and amplification of chondrocytes has been extensively studied in tissue engineering (Chesterman and Smith, 1968). Studies were mostly focused on the transplantation of isolated chondrocytes into cartilage defects. In 1994, Brittberg et al. (1994) described the autologous chondrocyte implantation (ACI) approach, which has been continuously developed since its first clinical use for articular cartilage repair. During the ACI procedure, chondrocytes were isolated using trypsin and col-lagenase digestion and cultured *in vitro* in DMEM/F12 with 10% autologous serum supplement. The culturing approach was originally established for the implantation of cells in suspensions, with periosteum or collagen membrane as the mechanical covering. Subsequent studies have improved the technique of cell culturing with a carrier membrane or a 3D scaffold. For instance, hyalograft-C biomaterial scaffold was constructed by network fibers with different-sized interstices, which can be employed as a 3D-scaffold to support cell proliferation, redifferention and extracellular matrix deposition with desirable mechanical properties (Gobbi et al., 2006). Research have also been focused on different phenotypic zones of cartilage tissue. Chondrocytes from the superficial, middle, or deep zones of articular cartilage can be harvested through surface abrasion technique, and then cultured in alginate and seeded on scaffolds to generate cartilaginous tissues. The mechanical and functional properties of cartilaginous tissues formed by different layers of chondrocytes exhibit varied results, but in clinical practice, chondrocytes are often extracted from a full-thickness biopsy (Brittberg et al., 2010).

In 2001, the concept of chondrocyte quality control was presented, with the idea that an enhanced stable population of chondrocytes could be employed to provide more consistent results from the transplantation of autologous chondrocytes. According to current research, approximately 100–150 million chondrogenic cells are required to generate an adult human auricular-shaped cartilage based on the type and porosity of the scaffold material (Bichara et al., 2012; Zhang et al., 2014). In addition, chondrocytes from the original pathologic auricle of patients can be extracted via enzymatic digestion, which suggested a possible treatment for microtia based on autologous auricular chondrocyte harvest. For example, He et al. (2020) cultured dedifferentiated microtia chondrocytes in a three-dimensional chondrogenic culture system and generated redifferentiated microtia chondrocytes, with the potential to regenerate mature cartilage.

However, major challenges can lie in the *in vitro* expansion process in which chondrocytes are prone to dedifferentiation, the determination of chondrocyte type and quantity, and the rapid loss of cartilage regeneration ability. To enhance the quality of engineered cartilage structures, several chemical stimuli, including collagen cross-linking boosters, growth factors, and catabolic enzymes have been used in regenerative medicine for cartilage-related abnormalities (Phull et al., 2016).

#### 2.1.2 Stem cells

Stem cells are also important cells with multilineage differentiation potential, which may be employed as a cell source for cartilage tissue engineering. For tissue-engineered articular cartilage, it is mainly implanted into the articular cavity that is rich in chondrogenic inducing factors such as cytokines and mechanical stimulation, which helps the implanted seed cells to fabricate mature chondroid tissue as well as retain the cartilage phenotype. Therefore, a variety of candidate seed cell sources have been accessible for further exploration. While for auricular cartilage reconstruction, there is a lack of chondrogenic inducing signal in subcutaneous transplantation area, which can be adverse to the phenotype stability of the cartilage generated by stem cells, resulting in fibrosis or ossification of chondrocytes (Liu et al., 2008). The current progress of stem cells in articular cartilage tissue engineering may provide reference value, and the co-culturing of chondrocytes and MSCs may also provide a more feasible scheme for seed cells to produce stable regenerated cartilage, which will be described in following section.

Mesenchymal stem cells (MSCs) can be isolated from adipose tissue, synovium, bone marrow and umbilical cord matrix, among which bone marrow-derived MSCs (BMSCs) are especially characterized with high proliferation rate and the potential for chondrogenic differentiation under proper tissue conditions (Moretti et al., 2010) and certain chondrogenic inducing factors such as insulin growth factor 1 (IGF-1), transforming growth factor β1 (TGF-β1) and platelet-rich plasma (PRP) (Mara et al., 2010; Xie et al., 2012; Maldonado and Nam, 2013). Moreover, the multilineage differentiation potential could be maintained after multiple amplification, which has made BMSCs a conductive choice to achieve tissue-specific repair of articular cartilage defects (Zhou et al., 2004; Zhou et al., 2006). Studies have also demonstrated the therapeutic potential of these cells in osteoarthritis (Cho et al., 2020; Anil et al., 2021). Although promising results have indicated the potential as a novel cell source for cartilage repair procedures, the main drawbacks of employing BMSCs are the invasive approach for harvesting cells and the possible complications (Urlić and Ivković, 2021). In addition, it has been shown that the frequency of BMSCs is relatively low (0.001%–0.01%) and that their differentiation capability decreases with age, which may impair the effectiveness of BMSCs in stem cell treatment (Zaim et al., 2012). Moreover, the engineered cartilage regenerated from MSCs was found to present more significant genetic differences from natural cartilage compared to that from primary culture chondrocytes, which may limit the clinical application of MSCs (Bomer et al., 2016).

Adipose-derived stem cells (ADSCs) can differentiate into adipocytes, osteocytes, chondrocytes, and nerve cells under different conditions. These cells have the advantages of abundant tissue sources, easily accessible sampling, low immunogenicity, rapid proliferation and multi-directional differentiation potential, which has become a research hotspot as seed cells in cartilage tissue engineering (Fahy et al., 2018). TGF-β has been proven to induce chondrogenic differentiation of ADSCs (Cicione et al., 2015). IGF-1 was also reported to have the potential alone or act with TGF-β1 to induce chondrogenic differentiation of ADSCs *in vitro* and *in vivo* (Lee et al., 2013). Griffin et al. (2019) utilized an argon plasma to modify the nanocomposite polyurethane scaffolds, which were found to facilitate the osteogenic and chondrogenic differentiation of ADSCs.

Induced pluripotent stem cells (iPSCs) can be obtained from somatic cells through reprograming procedures, which were firstly derived from retrovirus-mediated transcription factors into fibroblasts and eventually achieve embryonic-like multilineage differentiation potential (Diederichs et al., 2019) (Takahashi and Yamanaka, 2006). Independent studies considering chondrogenic differentiation application have been focused on osteoarthritis (OA) models. iPSCs were proved to differentiate into chondroid cells *in vitro* under induced directional induction and successfully generate articular matrix cartilage to repair defects after transplantation (Zhang et al., 2016; Zhu et al., 2016; Rim et al., 2018). A study utilized 3D bio-printing Kenzan approach to fabricate a scaffold-free engineered cartilage construct based on iPSCs. Anatomically shaped cartilage constructs were produced with improved mechanical strength, which may provide a clinically translatable strategy for chondral resurfacing in articular cartilage damages (Nakamura et al., 2021). However, uncertainties of the genetic modification remain to be evaluated, including the target gene overexpression, tissue malformations and impact on pathogenicity and safety of the host, which may be future challenges for iPSC-based cartilage reconstruction application (Yamashita et al., 2018).

Although articular cartilage tissue is not intrinsic reparative, it has demonstrated the existence of progenitor cells that promote the appositional growth of the tissue. Articular cartilage stem cells/progenitor cell (ACPC) was first identified and isolated by Gary P (Dowthwaite et al., 2004). from the surface zone of articular which possesses multilineage differentiation potential, with chondrogenic predisposition in particular. Subsequent research and phenotypic analyses illustrated that ACPCs may serve comparable functions to other tissue-specific stem cells (Fickert et al., 2004; Hiraoka et al., 2006; Seol et al., 2012; Bernstein et al., 2013; Jiang and Tuan, 2015). Jiang et al. (Jiang et al., 2016) group have identified a group of endogenous stem/progenitor cells derived from mature human chondrocytes by tracking the specific molecular markers, which were defined as chondrocyte-derived progenitor cells (CDPCs). CDPCs showed similar phenotypes as BMSCs, while exhibiting greater chondrogenic potential. Furthermore, CDPCs were used to treat large-scale cartilage tissue defects in clinical practice. All fifteen patients who received tissue-engineered cartilage tissue transplantation based on CDPCs achieved satisfactory recovery. The discovery of CDPCs may provide a promising prospect for clinical application. A study involving ACPCs, MSCs and chondrocytes reported that based on platelet-rich plasma (PRP) gel scaffold, ACPCs showed superiority in migration, proliferative and chondrogenesis capacities over the other cell types, which indicated a new strategy for cartilage regeneration (Wang et al., 2019). Meanwhile, ACPCs have also been discovered in both healthy and microtia ear remnants. Evaluation of the repair capabilities via *in vitro* culture revealed that these cells possess a robust ability to proliferate without losing their multipotent differentiation capacity and to create cartilage-like matrix in the culture framework (Otto et al., 2022), which may suggest a feasible candidate cell type for ear reconstruction.

## 3 Materials for biological scaffolds

The biological scaffold serves as a temporary substitute that mimics the cartilage extracellular matrix, on which the isolated seed cells can be planted after *in vitro* expansion. Subsequently, after *in vitro* culture, they are transplanted back into the body to achieve the purpose of tissue repair or replacement (Huang et al., 2018). The ideal scaffold should preserve a structurally stable three-dimensional projection, satisfactory biocompatibility and degradability, and be easy to manipulate into a certain form. It should be able to provide a favorable physiological environment for the cells while also maintaining the temporary mechanical integrity that is necessary to generate an elaborately structured framework throughout the process of chondrogenesis. Meanwhile, to sustain the form of the construct, the degradation rate of the scaffold must be commensurate with the tissue regeneration rate. In addition, it should be sturdy while providing flexible structural support to complement the neocartilage (Hoshi et al., 2018; Yang et al., 2019). Over the past few decades, varied types of natural, synthetic and hybrid materials have been explored *in vitro* and *in vivo* as biological scaffolds (Pearson et al., 2017).

### 3.1 Natural materials

For auricular tissue engineering, natural materials are mainly hydrogel polymers, among which alginate, pluronic, hyaluronic acid, chitosan, and collagen derivatives have been investigated as potential candidates to produce bioactive scaffolds (Yue et al., 2015; Rastogi and Kandasubramanian, 2019). The capacity to be injected and shaped into three-dimensional structures is an evident benefit of these materials. Besides, no apparent cytotoxicity has been found and the inflammatory reaction can be mild. However, inadequate mechanical strength, fast deterioration rate *in vivo*, and difficulties in morphological maintenance are their main drawbacks (Catoira et al., 2019; Yang et al., 2019; Salehi et al., 2020).

Alginate is derived from seaweed and forms hydrogels when in contact with calcium ions. The physical characteristics can be modified by adjusting the concentrations of alginate and calcium, which has been utilized to preserve chondrogenesis in animal and human and produce neocartilage (Ponticiello et al., 2000; Chang et al., 2001; Chang et al., 2003). Leslie et al. (2013); Leslie et al. (2018) developed a method to obtain degradable alginate saline gel by adding alginate lyase which can deliver stem cells through injection. The controlled release of ADSCs was achieved through alginate microbeads, which were then confirmed to form the chondroid tissue in rabbit ear defect model. Gelatin can be obtained through partial hydrolysis of collagen. When added with β-fibroblast growth factors (β-FGFs), the gelatin sponge scaffold can promote a slow release of β-FGF to facilitate the formation of auricle cartilage (Otani et al., 2015). The interaction of thrombin and fibrinogen yields fibrin gel which can be extracted from autologous plasma, and it can also be employed as a vehicle for cell transport (Silverman et al., 1999). Yue et al. (2021) developed a chondrocytes/chondrocyte-microtissues laden fibrin gel ear-shape scaffold, which has regenerated a stable anatomical structure in a rabbit model. Pluronic F-127 (consisting of 70% polyethylene oxide and 30% polypropylene oxide) is a synthetic thermosensitive hydrogel that has been illustrated to sustain engineered neocartilage (Cao et al., 1998; Saim et al., 2000; Kamil et al., 2004). Research directions have also focused on the modification of natural polymers via cross-linking and surface functionalization to enhance the mechanical and degradation properties (García-López et al., 2015; Xu et al., 2020). Collagen gel sponges were believed to increase the adhesion and proliferation of chondrocytes and the production of fibrocartilage-like ECM (Houck et al., 2018). More recently, studies based on collagen gel were carried out which focus on the mixture of seed cell types with different ratios (Cohen et al., 2018) and appropriate times of the seed cell passage (Bernstein et al., 2018), indicating that the tissue engineering ear reconstruction technology is developing towards a more precise and optimized strategy.

### 3.2 Synthetic materials

Synthetic polymers, notably aliphatic polyesters with fine biodegradability and biocompatibility such as polyglycolic acid (PGA), polylactic acid (PLA), and poly-caprolactone (PCL) and are widely used as medical biodegradable materials, controlled drug delivery systems and tissue engineering scaffolds (Arif et al., 2019; Wu et al., 2021). The advantage that synthetic polymers own over biological polymers is that they are custom-made materials whose biological and material features may be modified chemically and physically, and thus ensures strong plasticity, fine mechanical properties, and accurate control of morphology maintenance after implantation (Chen et al., 2020a). However, foreign body response and the lack of surface morphology that promotes cellular adhesion and development are the main limitations. The substance and its degradation products can trigger the foreign body inflammatory response and inhibit cartilage regeneration after implantation (Coenen et al., 2018). In addition, the lack of physiological qualities of flexible materials, material exposure, infection, and other complications might also be potential concerns.

In 1997, Cao et al. (1997) reported the generation of human ear-shaped engineered cartilage in a nude mouse model which consisted of a PGA/PLA scaffold seeded with bovine chondrocytes. After removing the supporting stent, the constructs demonstrated instability and deformation, but the picture vividly demonstrated the enormous clinical translation potential of tissue engineering. Shieh et al. (2004) assessed PGA scaffolds covered with poly-L-lactic acid (PLLA), PCL, and poly-4 hydroxybutyrate for engineered cartilage formation and structure maintenance in nude mouse and rabbit models for 10 months. Cartilage formation was observed in scaffolds of all types with the PCL obtained with the optimal structure of human auricular, while constructions showed significant deformation in rabbit models. A number of follow-up studies utilizing different types of cells and combinations of synthetic polymers have been published since then. Nakao et al. (2017) constructed nanoscale-diameter PGA scaffolds with the microtia remnant chondrocytes seeded on and found the regenerative cartilage in histological similarity to normal auricular chondrocyte, which maintained over 40 weeks. Zhang et al. (2014) cultured microtia chondrocytes and BMSCs with pressed PGA fiber mesh coated with PLA scaffold. The ear-shaped construction showed a complex structure with 100% chondrocytes formed. In 2018, Zhou et al. (2018) conducted the regeneration of patient-specific auricular and the first clinical translation in the field of tissue engineering in total ear reconstruction. In this study of five unilateral microtia patients, a three-dimensional (3D) printed resin model was employed to fabricate a scaffold comprising PCL as the kernel and PGA/PLA for the outer layer. The scaffold was trimmed according to the resin ear model, implanted with ear chondrocytes and cultured *in vitro*. The first patient presented a satisfactory reconstructed auricular morphology after the 2.5-year follow-up, and the histological examination also showed typical cartilage formation which resembles natural cartilage. Meanwhile, they also preliminarily coped with the inflammatory reaction of stent material by extending the induction time *in vitro*. This research provided a new strategy to improve the mechanical strength of engineered cartilage and maintain the morphology after implantation. However, the residual polymer materials are prone to cause aseptic inflammation, and the formation and distribution of chondrocytes and ECM also affect the mechanical stability, which remains the major problems to be solved in the application of synthetic material scaffolds.

### 3.3 Hybrid materials

During the last decade, hybrid materials consisting of natural and artificial synthetic materials have gradually become a research hotspot on account of the shortcomings of single materials. Experiments have been carried out to obtain composites with good histocompatibility, controllable morphology, and appropriate mechanical properties with the fabrication of hybrid implants. There are also studies emphasizing the combination of degradable and nondegradable materials in an attempt to help maintain the framework of the complex auricular contours.

Studies have reported the usage of fibrin gel where auricular chondrocytes are suspended to cover the Medpor framework with oxidizing solution utilized to alter the implant’s surface by adding hydrophilic properties (Lee et al., 2011; Hwang et al., 2014). There was a significant reduction of skin necrosis, implant exposure and extrusion with engineered cartilage-covered implants compared to the bare Medpor scaffold, which suggested a promising prospect of this combined implant with structural and functional stability for total ear reconstruction. Bichara et al. (2010) described cartilage engineering with alginate hydrogel and porous poly (vinyl alcohol). Improved surface chemistry and cartilage development on porous polyethylene may contribute to alleviating the constraints of scaffold extrusion and skin erosion. Alginate/PCL composite scaffolds were constructed with a 3D-printed PCL outer model and an injected alginate hydrogel, providing suitable mechanical and biomimetic properties for chondrocyte formation. With a pore size of 300 μm, the PCL model can be convenient for assembly, degradation and absorption (Visscher et al., 2019). A co-culturing of ADSCs and chondrocytes based on an alginate/PCL framework also depicted an enhanced tendency of chondrogenic differentiation (Jang et al., 2020). Similar results were seen in the co-culture of ADSCs and auricular chondrocytes by replacing the internal hydrogel with hyaluronic acid-collagen (Zopf et al., 2018). Hybrid materials composed of synthetic and natural biological materials in different forms or proportions can achieve comprehensive properties by improving the biocompatibility while also maintaining the mechanical properties, which may become preferable scaffold sources in future research and application of auricular tissue engineering.

## 4 Construction of biomaterial scaffolds of auricular mold

### 4.1 Scaffold fabrication techniques

Diverse biomaterial scaffold fabrication techniques have been established for processing different microstructures with controlled characteristics such as pore size, porosity, and pore interconnectivity, such that they are suited for chondrocyte growth, adhesion, and rapid nutrient transport (Kuberka et al., 2002). The most common methods include solvent casting/salt leaching, 3D fiber deposition, electrospinning, hydrogels and so on.

Solvent casting and particulate leaching (SC/PL) approach requires the construction of a salt/polymer suspension that is cast using a specific solvent. Upon solidification, as a result of solvent evaporation, the salt may be dissolved out of the scaffold and exit the pores. The pore spaces involved in such scaffolds may improve the proliferation and extracellular matrix production and benefit the growth of chondrocytes. However, this SC/PL technique was reported to achieve well effect mainly in small templates (Lee et al., 2003; Gong et al., 2008). Jing et al. (2006), (Wu et al., 2005) conducted a fabrication of 3D porous scaffolds based on a specially designed rigid-flexible mold using a particle leaching approach combined with compression molding. Scaffold shrinkage was tolerable under normal fabrication conditions with high salt contents, however, it requires precise control of the processing temperature and comparatively high loading of the compressing machine. Electrospinning can produce nanoscale fibers in an electrostatic field, resulting in high cell attachment-specific surface areas. These fibers resemble the collagen fibrils present in ECM, giving a highly porous, mechanical, and structural support. The usage of such scaffold architectures in cartilage tissue creation has shown encouraging results (Min et al., 2004; Li et al., 2005). Three-dimensional fiber deposition employs thermoplastic polymers that are delivered from a computer-controlled syringe onto a template so that the fibers can solidify in a predetermined manner, which is reproducible and revisable. As the layers of fibers accumulate, a structure of consistent pore size and 100% porosity can be formed (Woodfield et al., 2004).

### 4.2 Scaffold modeling techniques

Injection modeling has been extensively studied based on natural material scaffolds such as alginate (Chang et al., 2001; Chang et al., 2003; Dobratz et al., 2009), fibrin gel (Silverman et al., 1999) and pluronic F-127 (Saim et al., 2000). The engineered gel-chondrocytes construct can be molded by previously prepared silastic molds and injected into the subcutaneous tissue of animal molds (Yang et al., 2000) or used as a direct minimally invasive implant material for further exploration. However, the individual construction of such molds is time-consuming, and the quality cannot be precisely controlled and adjusted. The initial stage to construct an engineered auricle is the exact design and sculpting of the 3D distinctive contralateral auricle. Therefore, the development of computer-assisted processing methodology has become increasingly essential.

Computer aided design and manufacturing (CAD/CAM) has been reported in the preoperative planning and the creation of patient-specific ear prostheses (Nishimoto et al., 2014; Bos et al., 2015), and has also been utilized to assist in the fabrication of bioscaffolds in tissue engineering. Liu et al. (2010a) have developed an approach to precisely fabricate the auricular cartilage *in vitro* with the same structure which is mirror-symmetrical to the normal ear. The CAD/CAM method was applied to produce a negative cast of a half-sized human ear in a mirror image. Based on this mold, it was capable to form the PGA fibers into a scaffold in the shape of a typical ear in half size. Furthermore, they enhanced the mechanical strength of the PGA scaffold by coating it with an optimized amount of PLA, which as well maintained the biocompatibility of the framework. This technique has also been actively employed subsequently in the field of auricular tissue engineering (Zhou et al., 2018; Visscher et al., 2019; Jia et al., 2022).

### 4.3 3D printing techniques

Three-dimensional (3D) printing based on CAD/CAM enables rapid production of patient-specific anatomically attainable 3D models (Chae et al., 2015). Specifically, the CAD/CAM technique accurately carries out intricate 3D data transformations like scaling, mirroring, and Boolean operations. Printing methods can include extrusion, inkjet, laser-assisted, etc (Derakhshanfar et al., 2018). Spatial resolution and mechanical qualities may also be precisely controlled during 3D printing in addition to scaffold form (Gu et al., 2016; Mouser et al., 2020). The structures were constructed by layering biocompatible materials, known as cell-based “bio-ink,” from the bottom up (Zopf et al., 2015), and the expected tissues or organs can be obtained through *in vitro* culture. Bio-inks are cross-linked or stabilized during or immediately after printing to fabricate the desired structure (Kačarević Ž et al., 2018). An ideal bio-ink should provide tissue constructs with adequate mechanical strength and robustness while retaining tissue-matching mechanics and supporting chemical modifications in a specific tissue. It also requires fine biocompatibility, biodegradability, adjustable gelation and stabilization of the biomaterial (Loo et al., 2015).

Different natural and synthetic biomaterials with specific features have been identified as cell-laden bio-inks in different bioprinting applications (Lee et al., 2016a). Currently, there are two major types of 3D-printed auricle scaffolds, including hydrogels which auricle shape is printed directly, and biomaterials as support framework covered by a cell-containing hydrogel using 3D printing or immersing the scaffold into the hydrogel (Jang et al., 2020). Hydrogels have unique cell-binding sites that are advantageous for cell attachment, spreading, growth, and differentiation. In addition, several of these biomaterials may be readily photocross-linked in their modified forms (Wang et al., 2022). Silk fibroin (Rosadi et al., 2019), alginate (Unagolla and Jayasuriya, 2020), gelatin (Duan et al., 2013; Unagolla and Jayasuriya, 2020), and chitosan are often utilized as printing materials or used as part of a cartilage scaffold. Such hydrogels can act as a cell matrix to support cell growth (Unagolla and Jayasuriya, 2020). The use of high molecular weight polymers in 3D printing of irregularly shaped cartilage has also been reported in several studies, including poly (lactic-co-glycolic acid) (PLGA) (Wei et al., 2020), PLA (Rosenzweig et al., 2015), PCL (Xu et al., 2019; Li et al., 2021a), and polyurethane (Kim et al., 2019), to print cartilage scaffolds that are stable due to their optimal mechanical properties. Although natural bio-ink has good biocompatibility, its stability and mechanical properties are less satisfactory, and it is inclined to represent more rapid degradation, while synthetic bio-ink has excellent mechanical properties, but the lack of biological activity remains to be a major drawback (Wang et al., 2021).

Zopf et al. (2015) developed a 3D-printed PCL scaffold seeded with swine chondrocytes, which was then injected with a hydrogel-based construct involving growth and differentiation factors, which increased chondroinductivity in animal models after transplantation. Lee et al. (2014) constructed an auricular-shaped 3D scaffold comprised of cell-laden alginate hydrogel supported by a PCL-based biocompatible polymeric framework. This study utilized a method known as the Multi-head tissue/organ building system (MtoBS), in which two cytotypes were added to a three-dimensional construct independently. Chondrocytes and adipocytes derived from ADSCs were employed to generate auricular cartilage and the earlobe respectively and the chondrogenesis and adipogenesis were demonstrated by *in vitro* immunostaining (Lv et al., 2012). A unique 3D scaffold based on a chondrocyte-laden alginate construction with an integrated circular coil antenna attached to cochlear-shaped electrodes was designed by Mannoor et al. (2013). A study has designed a section of the polymeric framework with added silver nanoparticles, which were served as a conductive substance to embed the antenna. This attempt aimed to treat not only the cosmetic aspect of microtia but also the hearing impairment that results from it.

Gelatin methacrylate (GelMA) is a photosensitive semi-synthetic hydrogel that when combined with a photoinitiator, it can be quickly cross-linked and solidified to produce a three-dimensional structure of particular strength. The structure contains cell adhesion sites and matrix metalloproteinase hydrolysis sites, which enables cell growth and migration (Yue et al., 2015). The mechanical properties of the cross-linked hydrogel can be adjusted by changing the degree of substitution and the concentration of GelMA material. GelMA is mostly utilized for tissue engineering and 2D/3D cell culture due to its high biocompatibility. It can also be configured into mixed ink for 3D bioprinting based on the characteristics of printed tissue (Pepelanova et al., 2018).

In the field of auricular reconstruction, research has focused on the combination of PCL and GelMA as scaffolds, which depicts compressive properties similar to native auricular cartilage with satisfactory shape preservation, on which abundant cartilage-like matrix was produced based on progenitor cells (Otto et al., 2021). In another study, 3D-printed ear-shaped PLA scaffolds were prepared initially, and chondrocytes were fastened to the scaffolds via GelMA hydrogels, which showed good proliferative properties and stabilized structure after implantation (Tang et al., 2021). Novel bio-ink designs based on GelMA have also been actively conducted. A biomimetic microporous methacrylate-modified acellular cartilage matrix (ACMMA) was fabricated which then supported the generation of mature auricular cartilage-like tissues with satisfactory realistic form, elasticity and cartilage-specific ECM deposition *in vivo* (Jia et al., 2022). In combination with GelMa, bio-inks based on microtissues comprising microtia chondrocytes and cartilage acellular matrix (CAM) microparticles were printed by digital light processing with high printing accuracy. Mature cartilage regeneration was demonstrated in the mice model after transplantation (Xie et al., 2022).

Most recently, a first-in-human clinical trial of a 3D-bioprinted living tissue ear implant (AuriNovo™, United States) was conducted led by a regenerative medicine company (3DBio, United States) for ear reconstruction in patients with unilateral microtia (NCT04399239). The printed collagen hydrogel scaffold was made to encase the patient’s auricular chondrocytes after 3D scanning of the opposite ear which precisely matches the patient’s auricular shape. Exclusive 3D-bioprinter, bio-ink, cell culture system and implanted protective technology were designed systematically in the therapeutic production. This procedure may make breakthrough progress in the 3D bioprinting field in auricular reconstruction in the future, and the 3D-bioprinted implants may provide beneficial effects for microtia patients. Moreover, it also suggests the great significance of 3D bioprinting technique in a broader field of regenerative medicine, such as the realization of organ printing.

## 5 Inducing factors in stimulating chondrogenic differentiation and cartilage maintenance

It is estimated that approximately 100–150 million chondrogenic cells are required to generate an adult human ear-shaped cartilage (Bichara et al., 2012), but cells that have undergone different cultures may dedifferentiate and lead to the gradual loss of the original cartilage phenotype. Therefore, a variety of growth factors are required as crucial inducing elements which they can be incorporated into culture media directly or by other biological techniques to promote chondrocyte growth, morphology maintenance and cartilage formation. Currently, growth factors promoting cartilage regeneration in articular cartilage defects have been extensively studied, which also provides reference value and guidance for auricular cartilage reconstruction.

### 5.1 Growth factors

Transforming growth factor β (TGF-β) family act as multifunctional components mostly produced in cartilage and bone. TGF-β1, TGF-β2 and TGF-β3 are correlated with the processes of differentiation and de-differentiation of chondrocytes as a prelude to cartilage synthesis, stimulation of type II collagen and proteoglycans as well as differentiation of MSCs (Patil et al., 2011). TGF-β1 has been reported to facilitate inducing undifferentiated MSCs into a chondrogenic pathway, integrating chondrocytes into endogenous tissues and enhancing cartilage repair (Fan et al., 2006). The research of auricular reconstruction considering chondrocyte culturing has proved the vital role of TGF in improving redifferentiation and matrix formation of auricular chondrocytes, as well as proliferation and chondrogenesis of MSCs (Shieh et al., 2004). Bone morphogenetic proteins (BMPs) regulate the proliferation and differentiation of osteoblasts and chondrocytes (Katagiri and Watabe, 2016). Different types of BMPs have shown promising effectiveness in inducing chondrogenesis and auricular cartilage defect repair in animal models (Kuo et al., 2006; Vinatier et al., 2009; Li et al., 2021b). A study based on remnant auricular cartilage of microtia patients also illustrated that BMP-2 in the atelocollagen with the addition of insulin and T3 in the media could generate greater glycosaminoglycan (GAG) matrix in a shorter period but also sustain cell viability with lower mortality (Ko et al., 2012). BMP-7 and BMP-2 were also reported to help increase matrix production in nasal chondrocytes *in vitro* (Hicks et al., 2007). Insulin-like growth factor-1 (IGF-1) is considered a significant mediator mainly expressed in mature and developing cartilage which is involved in the maintenance of cartilage homeostasis (Davies et al., 2008). Studies have indicated that IGF-1 can serve as a stimulation factor for proteoglycan synthesis, chondrocyte proliferation, and cell homing in osteochondral defect repair (Pabbruwe et al., 2009; Cho et al., 2020). Combination of insulin and IGF-1 has shown additional benefits in formation and properties of engineered auricular cartilage with the thickness of native auricular cartilage (Rosa et al., 2014). Fibroblast growth factors (FGFs) are involved in chondrocyte proliferation, cell division and osteogenic processes (Itoh, 2014). FGF-2 was proven to increase GAG and type II collagen biosynthesis and proliferation and differentiation in costal chondrocytes and articular chondrocytes (Kato and Gospodarowicz, 1985; Veilleux and Spector, 2005), while FGF-18 was found to stimulate hyaline-cartilage production (Gigout et al., 2017; Sennett et al., 2018). Studies have also indicated that sustained release of b-FGF augments can enhance neovascularization and chondrogenesis in a tissue-engineered auricular cartilage construct (Isogai et al., 2005).

### 5.2 Other biological techniques

Despite the stable proliferation of auricular chondrocytes, dedifferentiation with rapid loss of chondrocyte phenotype and competence is unavoidable during repeated passages (Kang et al., 2012). The co-culturing system allows different cell types to be cultured together, which can help to investigate the effects and the molecules involved in cell-to-cell interactions such as cellular stimulation, gene pathways and cellular differentiation (Carter et al., 2015). Chondrocytes and MSCs were concurrently seeded onto an engineered scaffold in a co-culture experiment. It has shown that chondrocytes can stimulate BMSCs to differentiate into chondroblasts by producing exogenous growth factors, which can lessen the demand for exogenous growth factor supply (Fischer et al., 2010; Xue et al., 2012). Additionally, chondrocytes can act as a matrix for MSC migration and prevent MSC-derived chondrocytes from ossifying (Mo et al., 2009; Liu et al., 2010b). It has been proved that the biological induction co-culture mode of ADSCs and chondrocytes can induce the differentiation of ADSCs into osteoblasts and chondroblasts (Shi et al., 2017; Chen et al., 2020b). A study of a co-culture model of ADSCs and chondrocytes achieved successful production of cartilage based on 3D-printed bioresorbable scaffolds without the use of exogenous growth factors (Morrison et al., 2018). At present, research on co-culture of stem cells and articular chondrocytes has made significant progress in the field of biological engineering, which also provides guidance for auricular cartilage reconstruction. Co-culturing technique may develop a new solution for limited quantity and dedifferentiation of seed cells in cartilage tissue engineering and promote clinical translation of auricular cartilage engineering with lower cost and more stable tissue formation.

Genetic engineering technique intends to transfer cartilage growth factor genes into target cells through a carrier (such as adeno-associated virus) or attach the carrier to a biological scaffold so that the growth factor can achieve a stable and sustained expression to promote cartilage regeneration. Studies considering gene transfer of growth factors such as IGF-1, BMP-2, TGF-β and FGF-2 have been reported to lead to an enhancement in chondrogenic differentiation of MCSs (Cucchiarini et al., 2011; Lee et al., 2016b; Ikeda et al., 2017; Munsell et al., 2018). Genetic transfer technique has also become a research hotspot of cartilage, while more experimental evidence is still needed to evaluate the safety and effectiveness of this technology.

## 6 Conclusion and prospects

At present, surgical procedure based on autologous costal cartilage carving and transplantation remains the major treatment of total ear reconstruction. During the past decades, a variety of research and progress has been made in the field of auricular cartilage tissue engineering. The application of 3D printing and tissue engineering in medicine holds great promise for future innovation and more consistent results for microtia patients. The 3D printing approach can shorten operation time, avoid morbidity at the donor region, produce repeatable outcomes and reduce rejection rates compared to autologous costal cartilage transplantation. However, the prohibitive cost, limited application of printers and clinical translation obstacles are still confronted challenges before these potentially revolutionary choices become available to patients.

Clinical trials in human have also achieved preliminary results, but the safety and long-term effectiveness have not been fully confirmed. Before large-scale clinical application, a number of scientific and technical challenges need to be solved. The complex interrelation between cellular biochemistry, immunoreaction, and biomechanics of natural and synthetic material requires further investigation to design and fabricate the optimal individual-characterized auricular cartilage. The exploration and development of hybrid materials may be a favorable direction to obtain an ideal cartilage scaffold. The pathogenesis and molecular biomechanism of chondrogenic process in congenital microtia need to be further studied, which may contribute to the optimization of cartilage regeneration system.

The research progress of tissue-engineered cartilage in ear reconstruction requires mutual promotion of multiple research fields, and further to develop a systematic and standardized model of scaffold fabrication, cell extraction and culturing, and construct implantation to realize the clinical transformation and application of tissue-engineered auricular reconstruction in microtia.
